# Beyond donor shortage: tissue utilization and resilience in modern corneal transplantation

**DOI:** 10.3389/fmed.2026.1971505

**Published:** 2026-10-27

**Authors:** Ibtisam Ahmed Algouf, Hala Ali Daghriri, Mohammed Almutlak, Rafah Fairaq, Halah Bin Helayel

**Affiliations:** 1 Anterior Segment Division, King Khaled Eye Specialist Hospital, Riyadh, Saudi Arabia; 2 Eye Bank Department, King Khaled Eye Specialist Hospital, Riyadh, Saudi Arabia

**Keywords:** eye bank, eyebanking, keratoplasty, lamellar keratoplasty, tissue engineering

For decades, the global shortage of corneal tissue has been framed as a donor supply problem: increase donor numbers, increase transplants. While inadequate donation and local procurement remain major constraints in many settings, including countries that rely heavily on imported corneal tissue, procurement alone does not fully explain access to transplantation. Once donor tissue enters the transplantation pathway, preservation, allocation, logistics, surgical capacity, and recipient readiness also determine whether it ultimately reaches a patient. Corneal surgery has evolved far beyond the traditional model of penetrating keratoplasty. Lamellar keratoplasty, selective endothelial replacement, stromal augmentation, tectonic applications, and regenerative strategies have changed how tissue is used and how shortage should be understood. Donor tissue may be recovered yet not ultimately be used for clinical transplantation. Some non-utilization is unavoidable and appropriate, reflecting donor eligibility, tissue quality, microbiological or serological findings, or allocation to research and training. In other circumstances, potentially transplantable tissue may not reach a recipient because of logistical constraints, limited preservation time, case cancellation, or difficulties in timely reallocation.

Globally, an estimated 12.7 million individuals are waiting for a corneal transplant, with only one donor cornea available for every 70 patients in need (1). Importantly, the number of corneas recovered should not be equated with the number available for transplantation. Recovered tissue may subsequently be excluded because it does not meet clinical or safety criteria, including inadequate endothelial quality or findings identified during donor screening and microbiological or serological evaluation; tissue may also be allocated to research or training (2, 3). Therefore, recovery-to-transplantation ratios alone cannot quantify avoidable tissue non-utilization. A more informative assessment requires distinguishing appropriate exclusion from failure to transplant tissue that had already been deemed suitable for clinical use. The challenge is not only whether tissue exists, but whether it reaches the right patient, in the right form, at the right time. What ultimately matters is how effectively recovered tissue is preserved, allocated, repurposed when appropriate, and translated into clinical use.

Centers that depend heavily on imported tissue are exposed to these pressures. King Khaled Eye Specialist Hospital (KKESH) in Riyadh is a tertiary referral center serving patients from across Saudi Arabia. The role of the KKESH Eye Bank has evolved over time. Historically, KKESH served a broader national distribution role, receiving predominantly imported corneal tissue and supplying tissue to other centers in Saudi Arabia. More recently, several centers have established their own eye-bank services and obtain imported tissue directly according to their clinical needs. The KKESH Eye Bank now primarily supports transplantation within the institution through tissue receipt, storage, allocation, and coordination with surgical services, while maintaining a smaller local procurement pathway. Its role is also evolving toward more systematic and advanced local tissue processing. Although Descemet stripping automated endothelial keratoplasty (DSAEK) tissue has been prepared at KKESH for more than two decades, preparation was historically performed largely in the operating room according to individual surgeon preference. In 2025, a transition toward systematic DSAEK tissue processing within the Eye Bank using multiple technologies was initiated. This expansion also includes the development of an accurate, cost-effective laser-based method for local preparation of corneal allogenic intrastromal ring segments (CAIRS), with potential application to patch graft preparation. Femtosecond-laser technology is currently being implemented to further expand local processing capabilities, including CAIRS preparation, Bowman membrane transplantation, and DSAEK tissue preparation.

The vulnerability of import-dependent systems has been demonstrated during periods of major disruption. During the coronavirus disease 2019 (COVID-19) pandemic, corneal procurement and transplantation rates dropped sharply worldwide (4). More recently, the regional Middle East conflict beginning in mid-February 2026 disrupted supply chains at KKESH. Closure of major regional airport hubs required alternative shipping routes and adjustments to surgical scheduling. The surgical impact was measurable: in the second and third weeks of March, 100% of available surgical sessions were cancelled. Recovery was gradual, with only 25% of sessions proceeding in the first week of April and capacity fluctuating between 48% and 67% of normal levels in the following weeks. Pre-processed DSAEK and Descemet membrane endothelial keratoplasty (DMEK) grafts were particularly vulnerable because their procedure-specific preparation limited opportunities for reallocation. In response, pre-cut tissue orders were reduced, processing and surgical timing were adjusted to anticipated delivery, and priority was given to patients who could reach the hospital at short notice. This experience illustrates how quickly disruption in international transport can translate into reduced access to transplantation, even when donor tissue remains available elsewhere.

The evolution of corneal surgery has also changed how we think about the donor cornea itself. Historically, penetrating keratoplasty meant one donor for one recipient. Modern lamellar techniques have transformed that model: only the posterior layers are needed for endothelial keratoplasty, while anterior techniques leave posterior tissue untouched. A donor cornea can therefore be viewed as a collection of functional components rather than as a single graft.

Studies have shown that multiple stromal lenticules can be prepared from a single donor cornea, meaning one donation can help several patients (5). Lenticules obtained during small-incision lenticule extraction (SMILE) procedures, once routinely discarded, are being explored for therapeutic applications, including inlay and overlay additive keratoplasty and other ocular surface applications (6).

The uses of corneal tissue extend beyond optical keratoplasty. In glaucoma surgery, gamma-irradiated corneal tissue can be used for patch grafting over drainage devices (7, 8). Corneal tissue also has tectonic and reconstructive applications in other settings. In some cases, a single donor cornea can serve two patients: the endothelial component for keratoplasty and the remaining tissue as a glaucoma patch graft (9).

The versatility of donor corneal tissue continues to expand. Descemet membrane, harvested from corneas unsuitable for keratoplasty due to low endothelial cell counts or anterior stromal pathology, has been used as an epiretinal graft in patients with refractory full-thickness macular holes, with encouraging rates of anatomical closure and visual recovery (10).

Procedure-specific tissue preparation may reduce flexibility when the intended transplantation cannot proceed as scheduled because of patient-related, clinical, or logistical factors. In such circumstances, successful reallocation depends on identifying another suitable recipient within the remaining preservation window. At KKESH, patient scheduling and confirmation are primarily managed by the clinical services, whereas the Eye Bank coordinates tissue availability, allocation, and preparation with the surgical schedule. When transplantation cannot proceed as planned, the Eye Bank works with the clinical team to identify and contact suitable alternative recipients who can attend within the remaining preservation period. When feasible, surgery may be arranged outside the surgeon's originally allocated operating schedule, provided that both the patient and surgeon are available and agree to proceed.

Preservation strategy influences not only tissue viability but also the flexibility with which donor tissue can be allocated and transplanted. Hypothermic storage, commonly used in the United States and for imported tissue received at KKESH, provides a relatively simple preservation pathway but a limited storage window, generally approximately 7–14 days (3, 11, 12). This shorter interval requires closer coordination among tissue delivery, recipient readiness, surgeon availability, and operating-room access. Delays or cancellations occurring late in the preservation period may therefore leave less time for successful reallocation.

Organ culture, widely used by European eye banks, permits storage for approximately 4 weeks or more and allows microbiological surveillance before tissue release (3, 11, 12). This longer preservation window can facilitate tissue allocation, recipient selection, surgical scheduling, and reallocation when an intended procedure cannot proceed. Its principal advantage from a utilization perspective is therefore greater logistical flexibility rather than an increase in the underlying donor supply. However, organ culture requires dedicated infrastructure, controlled incubation and monitoring, microbiological testing, and additional tissue-handling procedures before transplantation (3, 11, 12). Thus, the relative advantages of hypothermic storage and organ culture extend beyond endothelial preservation to their effects on eye-bank workflow and the ability of transplantation systems to use available human tissue efficiently.

Glycerol-preserved and similarly processed tissues further expand the potential use of tissue that might otherwise expire, particularly in emergency and tectonic settings where optical clarity is not the primary concern (13). These approaches do not fully replace fresh tissue for optical keratoplasty but can provide additional flexibility during periods of system stress.

Tissue stewardship also extends to reducing the need for transplantation when effective alternatives are available. In keratoconus, earlier diagnosis and interventions that preserve or rehabilitate the native cornea may delay or avoid keratoplasty in appropriately selected patients. This represents another dimension of tissue conservation: reserving donor tissue for eyes in which transplantation remains necessary.

Looking ahead, biosynthetic implants, xenograft platforms, and gene-edited bioengineered corneas have shown promise and may eventually reduce reliance on human donors for selected indications (14–16). These technologies remain investigational, but they reinforce a broader shift: the future of corneal transplantation will depend as much on innovation in utilization and preservation as on donor recruitment itself.

More efficient use of available tissue does not diminish the importance of expanding the donor pool. In import-dependent centers such as KKESH, strengthening local corneal procurement remains a strategic priority. Expanding domestic procurement would improve resilience during supply-chain disruptions while complementing efforts to maximize the value of available tissue.

If corneal shortage is partly a problem of access and tissue utilization, the metrics used to evaluate eye-banking and transplantation-system performance should also evolve. Procurement and distribution volumes alone do not fully reflect how effectively available tissue is translated into patient care. Measures such as transplantation rates, appropriate versus potentially avoidable non-utilization, successful tissue reallocation, and use of different components of a donor cornea may provide a more complete picture. Integration of procurement, processing, allocation, surgical scheduling, and clinical outcome data could help identify barriers along this pathway. Practical measures, including coordinated patient readiness, backup-recipient pathways, flexible operating-room access for time-sensitive grafts, and redistribution between eye banks, may further improve utilization of transplant-suitable tissue.

The global corneal shortage will not be solved by increasing donation alone. The challenge is to ensure that eye-banking and transplantation systems evolve alongside surgical innovation: preserving tissue longer, using each donor cornea more effectively, anticipating disruptions to planned transplantation, and improving allocation and redistribution. Reducing potentially avoidable tissue non-utilization offers an opportunity to expand access without relying solely on donor recruitment.
